# Renal arteriovenous malformation: An unusual pathology

**DOI:** 10.4102/sajr.v23i1.1704

**Published:** 2019-05-30

**Authors:** Alain M. Mukendi, Amer Rauf, Sean Doherty, Florence Mahlobo, Peter Afolayan, Shabina Dawadi

**Affiliations:** 1 Department of Urology, Chris Hani Baragwanath Academic Hospital, University of the Witwatersrand, Johannesburg, South Africa; 2 Department of Radiology, Chris Hani Baragwanath Academic Hospital, University of the Witwatersrand, Johannesburg, South Africa

**Keywords:** Renal vascular malformations, congenital renal arteriovenous fistula, vascular nidus, coil embolisation, endovascular management

## Abstract

Renal arteriovenous malformations are rare renal vascular abnormalities. More commonly, the term refers to the congenital type of malformation. Only a few cases have ever been presented and reported in the literature, mostly with a nidus. We present the clinical, ultrasound and computed tomography findings and discuss the management related to a 63-year-old male with a right congenital renal arteriovenous malformation without a nidus that was successfully managed with coil embolisation. Relevant literature is hereby reviewed to highlight characteristic imaging and appropriate treatment.

## Introduction

Renal vascular malformations are abnormal communications between the renal arterial and venous systems. The most common type is acquired (arteriovenous fistula [AVF]), the congenital type occurring in less than 1% of the general population.^1^ Congenital renal arteriovenous malformations (AVMs) are rare renal vascular abnormalities and are a rare cause of haematuria which can be fatal.^2^

Based on the vessel architecture, congenital renal AVMs can be distinguished into three forms: the cirsoid type is the most common, with multiple arteriovenous interconnecting varix-like vascular communications; the angiomatous type, characterised by a single large artery feeding multiple interconnecting distal branches and draining veins; and the aneurysmal or cavernosal type, occurring in elderly patients, typically from a pre-existing arterial aneurysm which has eroded into an adjacent vein.^3^ In general, the congenital renal arteriovenous communication is made through a network of abnormal vessels (nidus) without interlaying capillaries.^4^

There are a limited number of reports in the literature regarding the topic of renal AVMs. We present the clinical, ultrasound and computed tomography (CT) findings and the management related to a 63-year-old male with a congenital right renal AVM, without a nidus, that was successfully managed with coil embolisation. Relevant literature is hereby reviewed to highlight characteristic imaging and appropriate treatment.

## Case report

**FIGURE 1:**
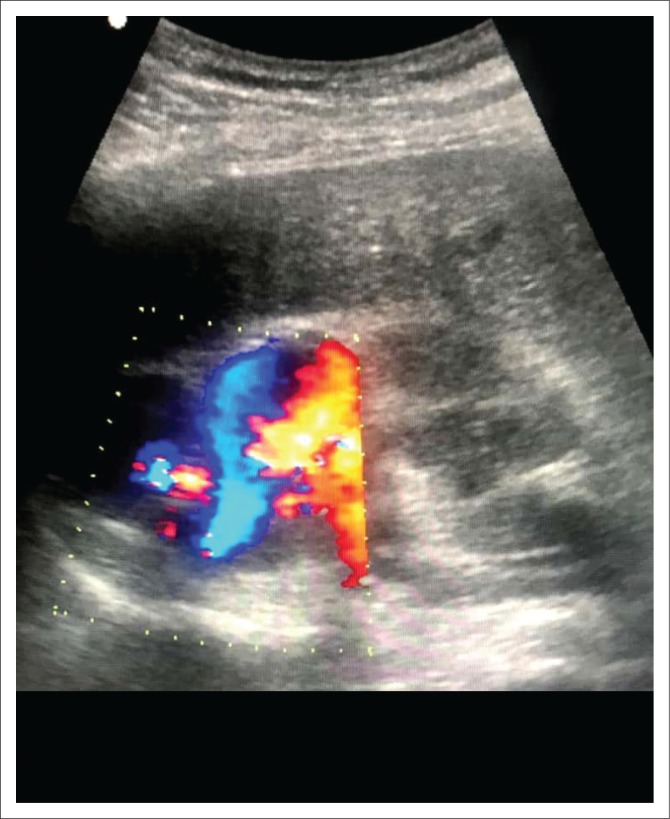
Ultrasound and Doppler study outlining a large, high-output arteriovenous communication in the upper pole of the right kidney.

**FIGURE 2:**
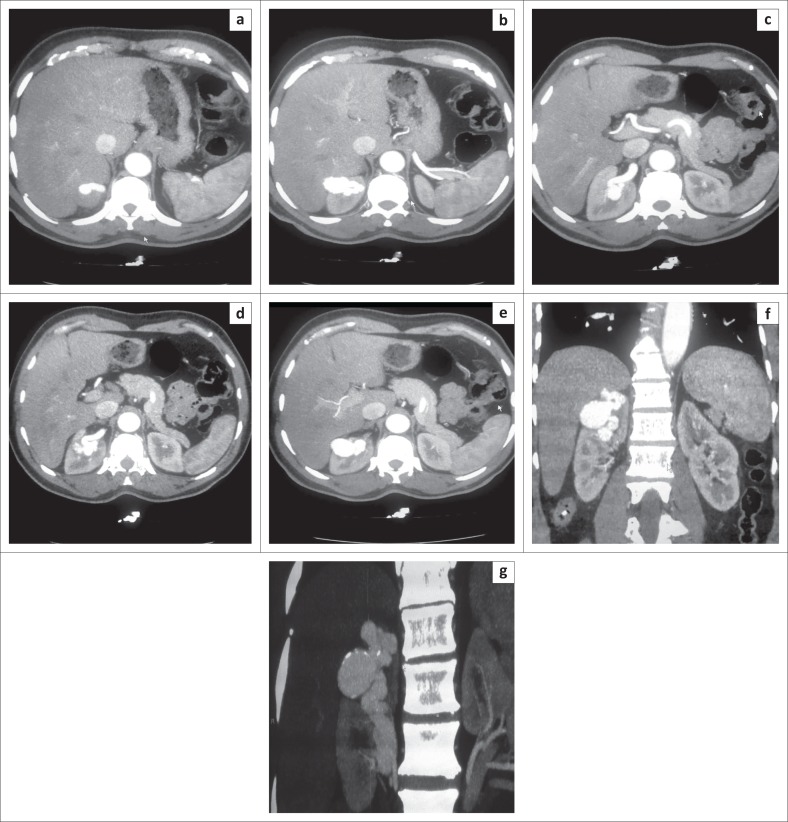
Computed tomography demonstrating the right upper pole arteriovenous malformation: (a–e) Axial plane images during arterial phase outline a single dilated upper pole segmental branch of the renal artery and immediate filling of the dilated draining vein; (f) Coronal plane images during arterial phase show a single dilated upper pole segmental artery and rapid filling of a single aneurysmal draining vein without any nidus; (g) Multi planar reconstruction in the coronal plane outlining the dilated right main renal artery and its upper pole segmental branch, a single dilated, tortuous draining vein and aneurysmal dilatation at the site of arterial communication.

A 63-year-old male patient was referred with lower urinary tract symptoms, who never had haematuria but suffered from episodic right flank pains that he described as pressure like pains. There was no previous history of renal trauma, renal biopsy or renal surgery. Physical examination was unremarkable. Ultrasound study outlined a large, high-output AVM at the upper pole of right kidney (Figure 1). Computed tomography angiography demonstrated enlargement of the right main renal artery, its anterior branch and the upper pole segmental artery of the anterior branch. An arteriovenous fistula was noted between a branch of upper pole segmental artery and a single draining vein. No nidus was seen. Ectasia of the feeding arterial segment and aneurysmal dilatation of the draining vein was seen at the site of the AVM (Figure 2).

**FIGURE 3:**
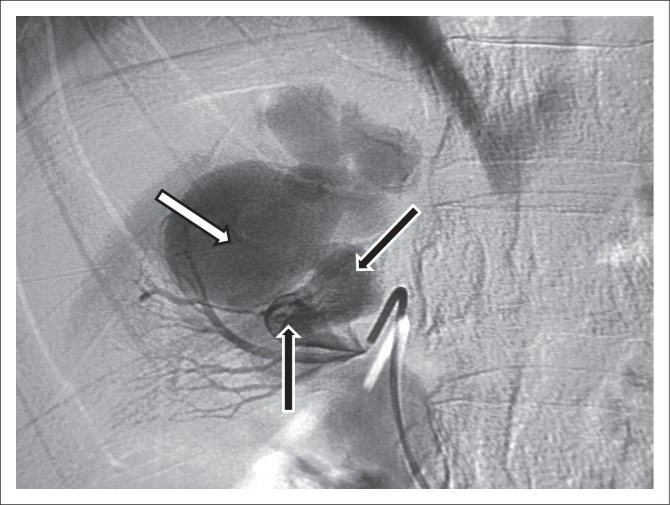
Angiogram demonstrating the arteriovenous malformation: The arterial feeder (black arrows) is shown communicating with the venous aneurysm (white arrow).

**FIGURE 4:**
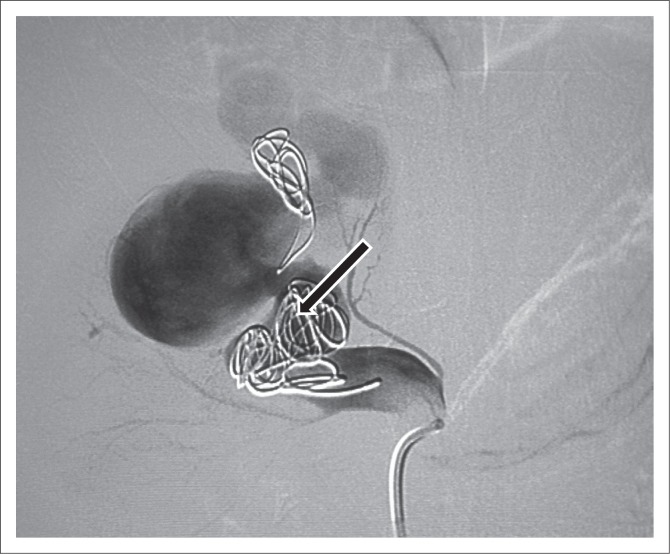
Angiogram after the first-coil embolisation demonstrating the first micro coil that migrated to the venous side because of high flow. The ectatic segment of the feeding artery was packed with coils but despite that there was still filling of the arteriovenous malformation because of high flow.

Endovascular management was the best option after weighing the risk versus benefit of a more invasive surgical option. Renal angiography was performed with a 5F C2 (cobra) catheter and angle tip 0.035” hydrophilic guide wire (Merit). A high-output AVM was outlined (Figure 3). A 0.018” guide wire and 2.8F micro-catheter were passed through a C2 catheter and positioned in the ectatic feeding artery of the AVM. The first micro coil slipped to the venous side because of high flow, but it did not migrate proximally and stayed close to the arterial feeder. Two micro coils were placed in the arterial feeder. The ectatic segment of the feeding artery was packed with two coils. However, the fistula was still filling due to high flow (Figure 4) and the procedure was abandoned because of incomplete embolisation with plans made for a second intervention.

**FIGURE 5:**
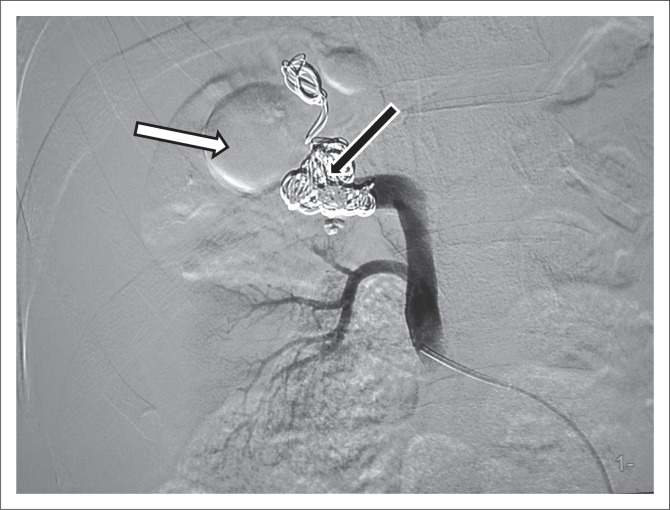
Angiogram after the second-coil embolisation demonstrating complete occlusion of the communication with coils.

At the second intervention, the empty spaces between the previously deployed coils in the feeding artery were packed with seven coils of different sizes. One small coil slipped into one of the branches of the upper pole segmental artery. Complete occlusion of the feeding artery was noted on the final angiograms. No filling of the venous channel was seen (Figure 5).

**FIGURE 6:**
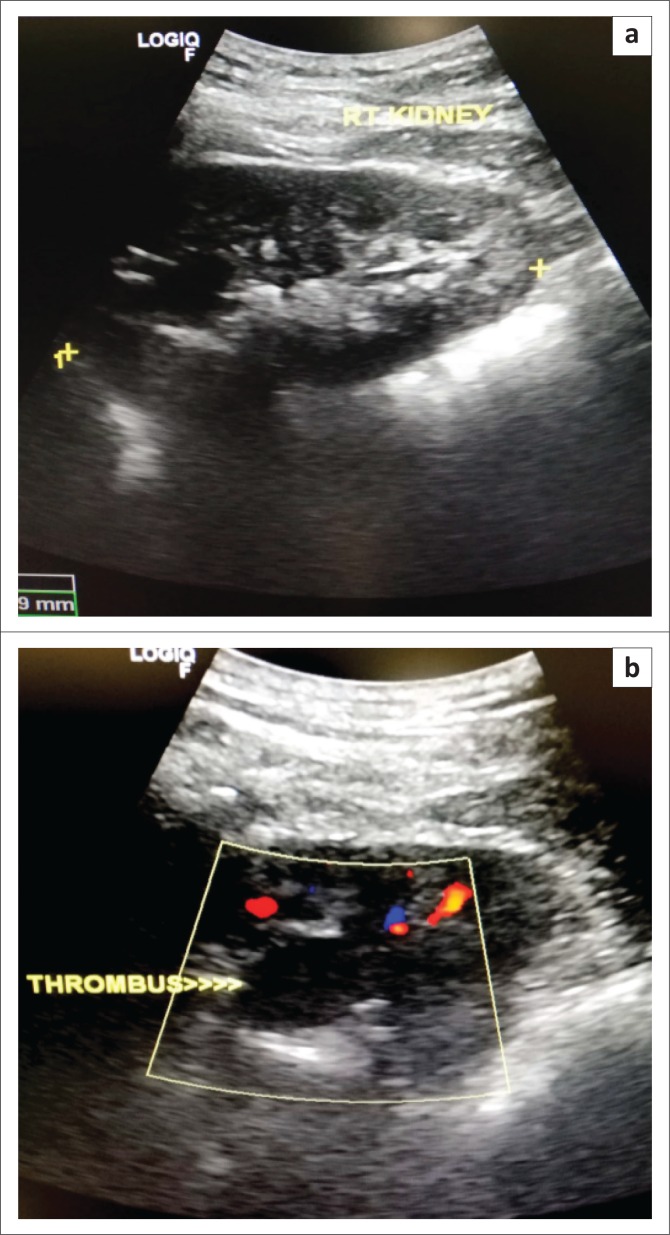
Post-embolisation Ultrasound and Doppler demonstrating a thrombus in the feeding artery and absence of venous flow 4 weeks after the procedure: (a) Post-embolisation Ultrasound of the right kidney showing a thrombus in the feeding artery at the right upper pole. (b) Post-embolisation Colour Doppler demonstrating absence of venous flow.

Following coil embolisation, no obvious post-embolisation syndrome (fever, nausea, vomiting and pains) was noted 48 h after the procedure. At the 2-week follow-up, renal Doppler demonstrated satisfactory occlusion of the AVM. The findings remained unchanged 4 weeks later (Figure 6).

## Ethical consideration

Human research ethics committee (medical) clearance certificate NO. M180894.

## Discussion

Renal AVMs can be congenital or acquired. As stated by Nassiri et al, renal AVMs are divided into two categories: acquired renal AVMs and congenital renal AVMs. The acquired types, commonly called AVFs, are the most common ones and are frequently caused by iatrogenic injuries such as renal biopsy or surgery, penetrating or blunt renal trauma and malignant renal tumours. The congenital renal AVMs are subdivided into two groups: (1) AVMs comprising multiple arterial feeders, a vascular nidus and multiple draining veins and (2) congenital renal AVFs (CRAVFs) which are rare (<3%), involving a single tortuous feeding artery and a dilated aneurysmal draining vein without a nidus.^5^ The patient presented in this report had a CRAVF.

The congenital type occurs as a result of focal spontaneous vascular development failures between the 4th and 10th week of life. They remain asymptomatic until the third or fourth decade of life.^6^ Patients most commonly present with haematuria resulting from rupture of small venules into the renal collecting system from raised intravascular pressure.^2^ About 30% of patients may present with signs of congestive heart failure from high-output fistulas, and up to 50% with cardiomegaly and hypertension.^7^ Other common clinical presentations include colicky pain, tenderness or fullness in the flank region and a continuous bruit over the flank.^8^

The two-dimensional mode ultrasound typically reveals a focal area of irregular connecting cystic lesions. Colour Doppler will demonstrate the presence of flow in these tortuous cystic areas, with a mosaic of colours indicating turbulent flow in these vascular malformations. Pulsed wave Doppler will reveal the presence of venous and arterial flow with high-flow velocity and turbulent diastolic flow. There is also spectral broadening present and pulsatile flow in the draining vein.^9^

Computed tomography plays an important role in the diagnosis of a possible underlying renal AVM. The correct scanning protocol, timed correctly, is essential to make an accurate diagnosis.^10^ A small renal AVM can be missed because of thickly sectioned images or inappropriate timing; therefore, thin-section CT using a collimation of at least 1 mm and a reconstruction interval of 0.5 mm–1 mm should be used. Multidetector CT with its high spatial resolution allows for evaluation of the shunt itself with CT angiography.^11^ Computed tomography findings include the following: pre-contrast – renal haemorrhage, renal parenchymal and vascular wall calcifications, round or oval masses in the collecting system which reveal themselves as aneurysms or varices on angiographic phase images; post contrast – early enhancement of the ipsilateral renal vein and IVC; CT angiography – dilated and tortuous arterial branches with aneurysmal dilatation of the feeding artery or draining vein, renal aneurysms or varices.^11^

Management of renal AVMs is either surgical or endovascular. Surgical management consists of partial or total nephrectomy. It is invasive, reserved for unstable patients or in patients with vascular anatomical difficulties. It requires several days of hospitalisation and is associated with high morbidity.^12^ Endovascular management for stable patients consists mainly of transarterial renal embolisation.^13^

Arteriovenous malformations without a nidus (as in this case) are mainly managed by blocking the feeding artery with coils or Amplatzer vascular plugs (AVPs). Metallic coils or micro coils of stainless steel or platinum are the main treatment option.^12,14^ Selection of the appropriate coil size and adequate packing of the arterial feeder are crucial for a successful procedure.

Coil migration is a known complication. Migrated coils in the venous system, such as the inferior vena cava, can be snared by retrieval sets. In high-flow fistulas, the risk of coil migration can be minimised by using detachable coils, inflating a balloon in the venous segment or putting an inferior vena cava filter (caging technique).^12^ Amplatzer vascular plugs are cylindrical self-expanding nitinol wire mesh.^15^ These are used in high-flow AVMs with aneurysmal dilatation of the feeding artery and high risk of coil migration.^5^

Arteriovenous malformation with nidus is treated with ablation of the nidus with gelfoam, alcohol and viscous liquid embolics. Coils cannot penetrate the complex network of a nidus, but liquid embolic ablation of the nidus can be combined with coil embolisation of arterial feeders in complex high-flow AVMs. Because of high flow, viscous sclerosing liquid embolic agents (NBCA [N-Butyl CyanoAcrylate], Onyx and Squid) are preferred over gelfoam and alcohol.

## Conclusion

Congenital renal AVFs are rare entities associated with a myriad of clinical presentations. Only a limited number of cases have been reported in the literature. Endovascular management is the treatment of choice for stable patients. Coils or amplatzer plugs are the main treatment option. Selecting the appropriate coil size for adequate packing of the arterial feeder or complete occlusion of the fistula is crucial for a successful procedure and avoidance of coil migration in high-output flow settings. In this case, complete occlusion was achieved and a follow-up Doppler test showed no flow.
